# Dynamic nonlocal metasurface for multifunctional integration via phase-change materials

**DOI:** 10.1515/nanoph-2024-0357

**Published:** 2024-10-10

**Authors:** Shilin Yu, Mingfeng Xu, Mingbo Pu, Xi Tang, Yuhan Zheng, Yinghui Guo, Fei Zhang, Xiong Li, Xiaoliang Ma, Xiangang Luo

**Affiliations:** National Key Laboratory of Optical Field Manipulation Science and Technology, Chinese Academy of Sciences, Chengdu 610209, China; State Key Laboratory of Optical Technologies on Nano-Fabrication and Micro-Engineering, Institute of Optics and Electronics, Chinese Academy of Sciences, Chengdu 610209, China; Research Center on Vector Optical Fields, Institute of Optics and Electronics, Chinese Academy of Sciences, Chengdu 610209, China; College of Materials Science and Opto-Electronic Technology, University of Chinese Academy of Sciences, Beijing 100049, China

**Keywords:** dynamic nonlocal metasurface, phase-change metasurface, bound states in the continuum, dynamic metalens, optical sensors

## Abstract

Non-local metasurface supporting geometric phases at bound states in the continuum (BIC) simultaneously enables sharp spectral resonances and spatial wavefront shaping, thus providing a diversified optical platform for multifunctional devices. However, a static nonlocal metasurface cannot manipulate multiple degrees of freedom (DOFs), making it difficult to achieve multifunctional integration and be applied in different scenarios. Here, we presented and demonstrated phase-change non-local metasurfaces that can realize dynamic manipulation of multiple DOFs including resonant frequency, *Q* values, band, and spatial wavefront. Accordingly, a metasurface integrating multiple distinct functions is designed, as a proof-of-concept demonstration. Utilizing the geometry phase of quasi-BIC and the tunability of vanadium dioxide (VO_2_), a dynamic meta-lens is achieved by tailoring spatial light response at quasi-BIC in the temperature range from room temperature to 53 °C. Simultaneously, the sharp Fano resonance of quasi-BIC enables the metasurface to serve as an optical sensor in the mid-infrared band, yielding a sensitivity of 7.96 THz/RIU at room temperature. Furthermore, at the metallic state of VO_2_ (80 °C), the designed metasurface converts into a mid-infrared broadband absorber, achieving higher than 80 % absorptivity and an average absorption of 90 % from 28.62 THz to 37.56 THz. The proposed metasurface enabling multifunctional performances in different temperatures can effectively improve the availability of devices and find more new and complex scenarios in sensing, imaging, and communications.

## 1 Introduction

Metasurface, a kind of artificial subwavelength planar digital optical device with thin thickness [1], [2], [3], has demonstrated an excellent ability to manipulate electromagnetic waves [4], [5], [6], [7], [8]. By exploiting the composite phase of dynamic phase and geometrics, asymmetric spin–orbit interaction and spin-decoupled functionalities can be realized [9], [10]. According to different generation mechanisms of light field response, metasurface can be regarded as two categories: local and nonlocal. The former manipulates light via individual meta-units, prototypically performing broadband optical wavefront shaping, such as phase-gradient metasurface [11], [12], [13], [14], Huygens metasurface [15], [16], plasmonic metasurface [11], [17], and catenary metasurface [18]. In contrast, nonlocal response is derived from kinds of interactions between adjacent metaunits. For example, photonic crystal slabs (PCS) upholding sharp Fano resonances [19], [20] are typical non-local metasurface. Such devices harness neighbor-neighbor interaction modes enabling accessible manipulation of optical spectra [21], [22], such as BIC metasurface [23], [24], [25], [26]. The local metasurface ignores optical interactions within meta-atoms, precisely the origin of non-local metasurface response, making it difficult to execute resonant spectral manipulation and spatial wavefront shaping simultaneously. Most recently, non-local metasurfaces with geometric phases enable exclusively wavefront shaping at a sharp quasi-BIC [21], [27], [28], [29], [30]. Such metasurfaces can simultaneously achieve high *Q* resonances and spatial wavefront shaping. Unlike BIC, an ideal state trapped with an infinite lifetime [31], quasi-BIC can be commonly obtained by destructive interference of structure or symmetry breaking [23], [32] in optical metasurface and extensively applied in nonlinear optics [33], [34], [35], sensors [36], [37] and laser [38], [39]. Quasi-BIC generated by introduced perturbations can manipulate the band structure and *Q* value, which makes non-local metasurfaces with geometric phases provide significant advantages of multidimensional regulation for multifunctional devices. However, quasi-BIC controls the band structure and *Q* values actualized generally by adjusting structural parameters and perturbations [23], [40], [41], which means a manufactured nonlocal metasurface lost the ability to manipulate multiple DOFs. Therefore, developing tunable and multifunctional device platforms in non-local metasurfaces remains a significant challenge, which can integrate highly differentiated functions into a compact metasurface and is promising to expand more new and complex scenarios.

Tunable and reconfigurable optical devices play a significant role in the future technology toward practical photonic devices [42], [43]. Different pathways have been demonstrated to attain tunable metasurfaces, including electromechanical tunability based on graphene [44], [45], nonlinear response [33], magnetically tunable metasurfaces [46], liquid crystal infiltration [47], and phase-change materials [48], [49], [50]. Among these methods, phase-change materials manifest the better pronounced and robust tunability of optical properties, and therefore, are extensively applied in tunable optical metasurface, including tunable metalens [51], free-space modulators [52], tunable Mie resonant nanoantennas [53], [54], dynamic information encryption [55], [56] and dynamic nonlocal metasurfaces [57], [58]. Particularly, VO_2_ is widely investigated [54], [59], [60]. A phase change can be triggered when the ambient temperature is close to 68 °C. Below the transition temperature, the optical characteristic of VO_2_ emerges as an insulator material. On the contrary, metallic properties are occupied dominantly. Therefore, it is a quick and simple way to accomplish the state switch between the insulator and metal state of VO_2_ by manipulating the ambient temperature, offering a promising solution to versatile and tunable meta-devices.

Here, we first presented and demonstrated the dynamic manipulation of multiple DOFs including resonant frequency, *Q* values, band, and spatial wavefront in phase-change non-local metasurfaces. And then, as a proof-of-concept demonstration, a metasurface integrating multiple distinct functions is designed. In the temperature range from room temperature to 53 °C (the insulator state of VO_2_), a dynamic meta-lens is achieved by tailoring spatial light response at quasi-BIC. Simultaneously, the sharp Fano resonance of quasi-BIC enables the metasurface to serve as an optical sensor in the mid-infrared band, yielding a sensitivity of 7.96 THz/RIU at 30 °C. Furthermore, at the metallic state of VO_2_ (80 °C), the designed metasurface converts into a mid-infrared broadband absorber, achieving higher than 80 % absorptivity and an average absorption of 90 % from 28.62 THz to 37.56 THz.

## 2 Results and discussion

**Figure 1:**
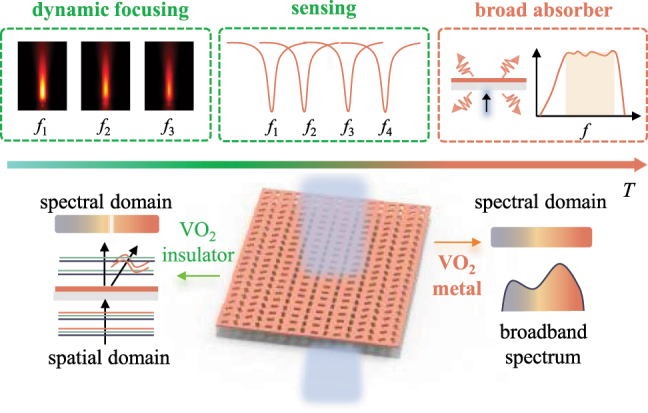
Conceptual description of multifunction-integrated phase-change nonlocal meta-surfaces. The gradient temperatures are described from green to dark orange, corresponding to the insulator and metal state of VO_2_, respectively. Within the temperature range of the VO_2_ insulator, the metasurface can simultaneously and independently manipulate high *Q* resonances (the white part) in the spectral domain and wavefronts (the orange curve) in the spatial domain, thus achieving the functions of dynamic focusing metalenses for frequency selection and refractive index sensors simultaneously. For the VO_2_ metal, the metasurface loses the ability of wavefront shaping and exhibits broadband spectrum characteristics in the spectral domain. Therefore, the metasurface has achieved the function of broadband absorbers.

The conceptual diagram of multifunction-integrated phase-change nonlocal meta-surfaces is displayed in Figure 1. Adjusting the ambient temperature can control the crystallization state of VO_2_, thereby achieving multifunctional integration in our designed metasurface. The gradient temperatures are described from green to dark orange, corresponding to the insulator and metal state of VO_2_, respectively. When the VO_2_ is an insulator, the metasurface can simultaneously and independently manipulate high *Q* resonances in the spectral domain and wavefronts in the spatial domain. With the changing temperatures, quasi-BIC is tunable and the geometry phase of quasi-BIC imparted by the converted circularly polarized (CP) light can tailor spatial light response only at quasi-BIC. Therefore, the proposed metasurface can simultaneously achieve the functions of dynamic focusing metalenses for frequency selection and refractive index sensors. Additionally, when VO_2_ is converted into a metallic state, the designed metasurface loses the ability of wavefront shaping and exhibits broadband spectrum characteristics in the spectral domain, thus realizing the function of broadband absorbers. The proposed metasurface is multifunctional and tunable with a compact and lightweight design, promising for multi-scenario applications such as dynamic wavefront shaping, mid-infrared imaging, optical refractive index sensing, dynamic tracking sensing, and broadband mid-infrared absorption.

### 2.1 Temperature-dependent manipulation of resonant frequency, *Q* factors, and band

**Figure 2:**
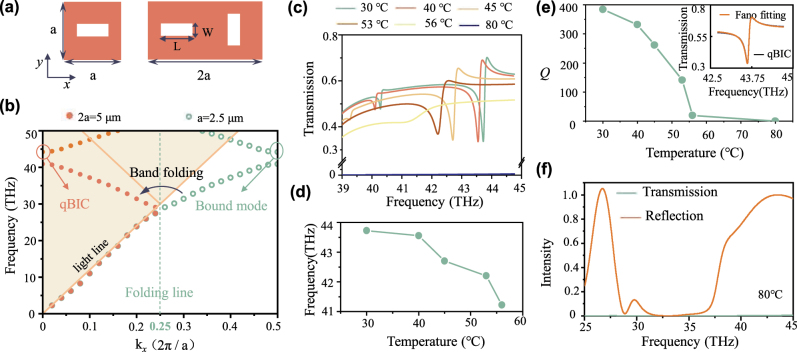
Formation mechanism and evolution of quasi-BIC in the phase-change nonlocal metasurface and their optical spectral properties. (a) The lattice and extended dimerized meta-atom with rectangular apertures (*L* = 2 μm, *W* = 0.6 μm) etched in a silicon thin film, where the period *a* = 2.5 μm, the thickness *t* = 0.8 μm. (b) Calculated band structures of single lattice and extended dimerized meta-atom, plotted with turquoise circles and orange solid dots, respectively. Band folding arises at the folding line, thus the bound state outside the light line is folded to the position of Γ point, forming a quasi-BIC. (c) Transmission spectra at different ambient temperatures when the LCP light is incident vertically. (d) Resonant frequencies of quasi-BIC at the corresponding temperatures. (e) Calculated *Q* values of quasi-BIC at different ambient temperatures and the inset shows the results of the Fano fitting. (f) The broadband spectra of the metasurface at 80 °C.

To uncover the dynamic manipulation process of multiple DOFs, we first interpret the formation mechanism and evolution of quasi-BIC in a phase-change nonlocal metasurface (as shown in Figure 2(a)). The BIC obtained in the dimerized metasurface is derived from the band folding caused by lattice expansion, thus called Brillouin zone folding-induced BICs (BZF-BICs) [24]. Figure 2(a) displays the lattice (the period *a* = 2.5 μm, the thickness *t* = 0.8 μm) extended along the *x* direction, wherein rectangular apertures (*L* = 2 μm, *W* = 0.6 μm) are etched in the VO_2_ thin film (material properties as shown in Figure S1). The calculated transverse electric (TE) band structures of the square lattice and extended lattice at room temperature are shown in Figure 2(b). Extended lattice leads to the folding of the Brillouin zone, where bound states originally located below the light line are folded into the Brillouin zone at the Γ point to form new bands. It leads to the transformation of the bound states that are not radiated to free space into the BZF-BIC. The BZF-BIC further transforms into quasi-BIC by introducing perturbation (*δ* = *L*−*W*). The evolution process of quasi-BIC with different perturbation values is provided in the Supplementary Materials (Figure S2). Therefore, by tailoring the perturbation values, it is possible to effectively manipulate the quasi-BIC including resonant frequency, band, *Q* value, and transmission. Furthermore, to dynamically control the DOFs, we investigated temperature-dependent manipulation of phase-change nonlocal metasurfaces.

We consider the phase-change metasurface working at different ambient temperatures. The quasi-BIC excited by the phase-change nonlocal metasurface can be effectively controlled by the external stimulus of temperature, achieving similar results to the manipulation by perturbations, as shown in Figure 2(c)–(e). The refractive indices of VO_2_ under different temperatures are provided in the Supplementary Materials (Figure S1) and the illumination is left circularly polarized (LCP) light. In the temperature range from room temperature to 53 °C (the insulator state of VO_2_), quasi-BIC resonance persists. Still, the resonance frequency undergoes a redshift (as shown in Figure 2(d)) and the *Q* value decreases continuously (Figure 2(e)). The *Q* value can be obtained by fitting the Fano formula [61] (Equation (1)).

(1) $$T\left(\omega \right)={\left|a_{1}+j{\;a}_{2}+\frac{b}{\omega -\omega_{0}+j\gamma }\right|}^{2}$$

where *ω*_0_ is the resonant frequency, *a*_1_, *a*_2_, and *b* are constant numbers, and *γ* is the overall damping rate of the resonance system. Here, *Q* = *ω*_0_/2*γ* and *γ* = *γ*_*i*_ + *γ*_*e*_, where *γ*_*i*_ and *γ*_*e*_ represent the intrinsic loss rate and the external radiation loss rate, respectively. The inset in Figure 2(e) shows the results of the Fano fitting. Under temperature stimulation, *γ*_*i*_ is controlled due to the varying refractive index of VO_2_, while *γ*_*e*_ remains unchanged. Therefore, the *Q* values of quasi-BIC can be effectively manipulated by the changing temperatures. Similarly, the resonant frequency, band, and transmission can also be tunable by the external temperature stimulation. Noteworthy, when the temperature is 80 °C, quasi-BIC disappears and the transmission is almost zero. The broadband spectra are displayed in Figure 2(f).

### 2.2 Dynamic manipulation of spatial wavefront

**Figure 3:**
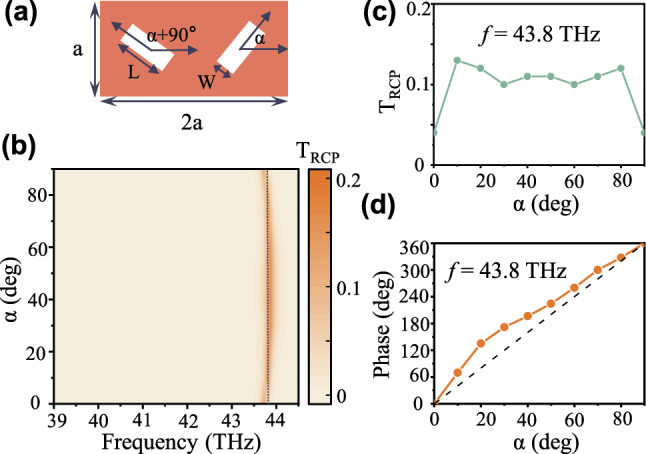
Demonstration of geometric phase relation for the designed non-local metasurface. (a) The dimerized meta-atom with rectangular apertures (*L* = 2 μm, *W* = 0.6 μm) etched in a VO_2_ thin film, where the period *a* = 2.5 μm, the thickness *t* = 0.8 μm. The rotation angle of the rectangular aperture is *α*. (b) Transmission spectra of RCP (*T*_rcp_) with different rotation angles *α*. The corresponding amplitude (c) and phase (d) under different rotation angles at the resonant frequency (*f* = 43.8 THz). Here, the short black line is the reference line of 4*α*.

Dimer meta-unit placed vertically as shown in Figure 3(a) can effectively support multiple geometric phases for cross-circular polarization light [21]. Under LCP light illumination, the geometric phase of different components in transmission can be written as

(2) $$\left\{\begin{array}{c} \Phi_{\text{rcp}}≃4\alpha \;\\ \Phi_{\text{lcp}}≃0\; \end{array}\right.$$

where *α* is the angle between rectangular holes and the *x*-direction, Φ_rcp_ and Φ_lcp_ represent the geometric phase of RCP and LCP light in transmission, respectively. At 30 °C, the transmission spectra of RCP (Trcp) with different rotation angles *α* are displayed in Figure 3(b), where the resonance remains steady at an almost fixed frequency. Figure 3(c)–(d) exhibit the corresponding amplitude and phase under different rotation angles at the resonant frequency (*f* = 43.8 THz). Simulated results manifest that the phase of *T*_rcp_ is the approximately linear 4*α* dependence of different rotation angle *α* from 0° to 90°, which is consistent with the theoretical predictions. The above meta-units compose a unit-cell library and enable a complete geometric phase ranging from 0 to 2*π*, supporting the construction of an arbitrary phase profile for wavefront shaping. This wavefront shaping only occurs at the frequency where quasi-BIC resonance emerges, so changes in the resonance position under temperature stimulation can lead to dynamic wavefront shaping.

### 2.3 Multifunctional metasurface and its performances

**Figure 4:**
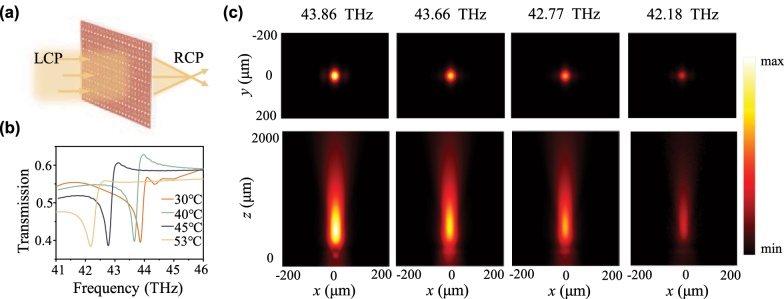
Design of a two-dimensional (2D) metalens based on the phase-change nonlocal meta-units and its performance for dynamic focusing. (a) Sketch map of the focusing metalens in 2D space. LCP light is normally incident and the transmitted RCP light is focused. (b) Transmission spectra of the designed metalens with different temperatures, where the resonant frequencies are 43.86 THz, 43.66 THz, 42.77 THz, and 42.18 THz, respectively. (c) The transverse and longitudinal far-field profiles through the focusing spot of the metalens under different temperatures for the converted RCP component at the corresponding resonant frequencies, 43.86 THz, 43.66 THz, 42.77 THz, and 42.18 THz, respectively.

Utilizing a library consisting of the dimerized meta-atoms with different rotation angles *α*, we design a two-dimensional (2D) metalens, achieving a narrow-band focusing as shown in Figure 4(a). Only the transmitted RCP light is focused under the normally incident LCP light. The archetypal phase profiles of the 2D metalens are described as

(3) $$\varphi \left(x,y\right)=\frac{2\pi }{\lambda_{0}}n\left(\sqrt{x^{2}+y^{2}+F^{2}}-F\right)$$

where $\varphi \left(x,y\right)$ indicates the phase at an arbitrary position (*x*, *y*), *λ*_0_ represents the working wavelengths, *F* and *n* are the focal length and the refractive index of the focusing medium, respectively. Here, the designed metalens performs an ideal focal length of 368 μm and the working wavelength is the same as the quasi-BIC resonant wavelength for the phase-change nonlocal metasurface. Meanwhile, the radius of the designed metalens is 75 μm. The designed metalens is coded by VO_2_ meta-atoms of the obtained library, leading to a space light focusing of the converted RCP light under normal incidence LCP light.

To demonstrate the dynamic focusing ability of the metalens, typical temperatures including 30 °C, 40 °C, 45 °C, and 53 °C are selected to analyze the focusing performance. At the different temperatures from 30 °C to 53 °C, the transmission spectra of the designed metalens are displayed in Figure 4(b), and the quasi-BIC resonances are obtained at 43.86 THz, 43.66 THz, 42.77 THz, and 42.18 THz, respectively. Compared with the results in Figure 2(c), almost the same resonant frequencies manifest that the designed metalens is fundamentally robust even though the meta-atoms are placed with different rotation angles. Such robustness implies that the spectral response of a nonlocal meta-device can be determined by only engineering a single meta-atom, thus greatly reducing the complexity of metasurface design. The transmission spectra of the converted RCP light with different temperatures are also displayed in Supplementary Materials (Figure S3), which can simultaneously describe the focusing efficiency of the designed metalens under different temperatures. The gradual decrease in transmission intensity of RCP light with increasing temperature is attributed to an increase in inherent losses (γi). Meanwhile, the working frequency of the metalens is tunable due to changes in ambient temperature, the converted RCP light is dynamically focused around different resonances according to different temperatures. The far-field profiles of transmitted RCP light in Figure 4(c) demonstrated the efficient focusing ability at the corresponding quasi-BIC frequencies with different temperatures. The weakening focused light intensity with increasing temperature is attributed to the decreasing intensity of RCP light, essentially stemming from the higher material loss of VO_2_. Additionally, the designed metalens can select the working frequency and the focus occurs only around the quasi-BIC resonance (as shown in Figure S4). Away from the resonance center, the focused intensity of RCP light can be ignored. Thus, as VO_2_ is the insulator state, with the temperature from 30 °C to 53 °C, the designed metasurface can be regarded as a dynamic metalens. This dynamic manipulation of the narrowband wavefront can find many applications in optical information processing, typically, dynamic mid-infrared imaging.

**Figure 5:**
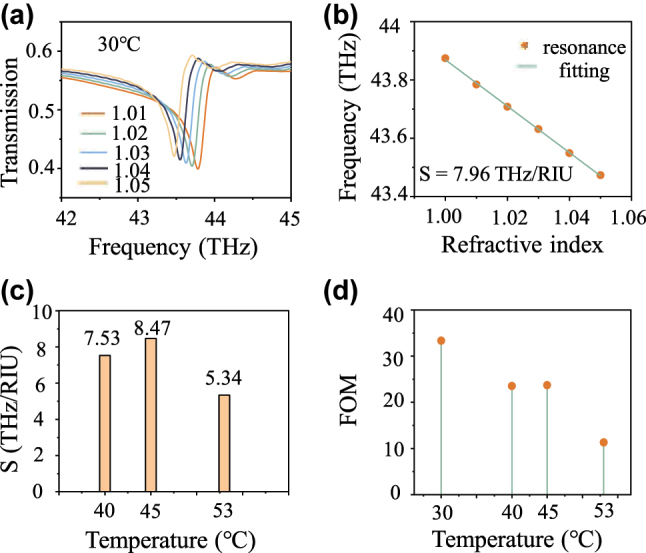
Sensing performance of the designed metasurface at the temperatures from 30 °C to 53 °C. (a) Transmission spectra of the designed metasurface under different temperatures. (b) The resonant frequencies of different ambient refractive indices at corresponding temperatures (the orange solid dots) and a fitting curve (the solid green line) at 30 °C, where the calculated sensitivity is 7.96 THz/RIU. (c) The corresponding sensitivities are calculated at 40 °C, 45 °C, and 53 °C, which are 7.53 THz/RIU, 8.47 THz/RIU, and 5.34 THz/RIU, respectively. (d) The FOM values are calculated at different temperatures from 30 °C to 53 °C, corresponding to 33.35 RIU^−1^, 23.52 RIU^−1^, 23.72 RIU^−1^, and 11.31 RIU^−1^, respectively.

Sharp Fano resonance of the quasi-BIC enables the designed metalens to serve as a refractive index sensor. The sensitivity of a refractive index sensor [36], [37] can be given by *S* = Δ*f*/Δ*n*, where Δ*f* and Δ*n* denote the frequency shift and change of the ambient refractive index, respectively. Transmission spectra of the metalens with different ambient refractive indices are displayed in Figure 5(a), where a redshift occurs for the quasi-BIC resonance. Figure 5(b) shows the resonant frequencies of different ambient refractive indices and a fitting curve, results illustrating the quasi-BIC of the designed metalens is linear with the change of ambient refractive index. And the calculated sensitivity is 7.96 THz/RIU. Sensitivity at other temperatures is also calculated, as displayed in Figure 5(c). The corresponding sensitivities at 40 °C, 45 °C, and 53 °C are 7.53 THz/RIU, 8.47 THz/RIU, and 5.34 THz/RIU, respectively. Results imply a relatively steady sensitivity performance of the designed metasurface. Figure of merit (FOM) is another key parameter to evaluate the performance of sensors [36], [37]. FOM can be obtained by FOM = *S*/Δ*f*, where *S* is the sensitivity and Δ*f* represents the frequency interval between resonance peak and valley. The calculated FOM values corresponding to different temperatures are displayed in Figure 5(d). The values of FOM reduce along with the increase in temperature. Such is caused by the increase in inherent losses of VO_2_, consistent with the results and analysis in Figure 2. Therefore, the designed metasurface performs the abilities of dynamic focusing and sensing at temperatures from 30 °C to 53 °C, meaning it can be utilized in very different scenarios. Additionally, multifunctional performance makes it promising for complex scenes, such as dynamic target tracking and cell ranging. In such scenes, the ability to focus can locate and image the target object while the refractive index of the target object or the distance from the target to the metasurface can be measured based on the sensing performance.

**Figure 6:**
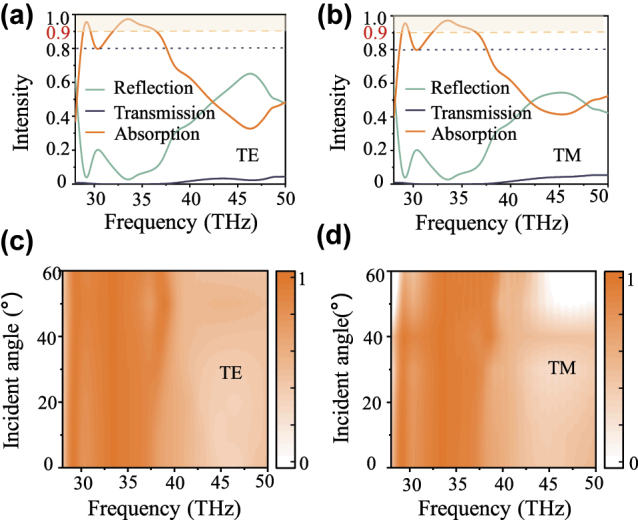
Broad absorber performance of the designed metasurface at 80 °C. The optical spectra of the designed metasurface for the normal incident TE (a) and TM waves (b) at 80 °C. The absorption spectra as a function of the incident angle for both TE (c) and TM waves (d).

In terms of the metallic state of VO_2_ at 80 °C, the change of material properties enables broad-band characteristics of the phase-change nonlocal metasurface, which can serve as a mid-infrared absorber. Herein, the absorption can be calculated by

(4) $$A\left(f\right)=1-R\left(f\right)-T\left(f\right)$$

where *R* and *T* represent the reflection and transmission of the designed metasurface at 80 °C. Figure 6(a) and (b) display the optical spectra of the designed metasurface at 80 °C, corresponding to transverse electric (TE) and transverse magnetic (TM) polarizations at normal incidence, respectively. For TE waves, the simulated results manifest that the absorption is above 80 % in the frequency range from 28.62 THz to 37.56 THz and the average absorption reaches 90 % in this band. Meanwhile, the absorption exceeds 90 % in the frequency bands of 28.83–29.62 THz and 31.87–36.57 THz. For the TM waves, in the frequency range from 28.6 THz to 37.58 THz, the absorption exceeds 80 % and an average absorption of 90 % is obtained. At normal incidence of TE and TM light waves, the performance of the available absorption part is almost the same. Meanwhile, the absorption spectra with different temperatures are also displayed in the Supplementary Materials (Figure S5). For the metal state of VO_2_, the absorption performance of the designed metasurface is independent of temperature. Further, we investigated the absorption spectra as a function of the incident angle for both TE (Figure 6(c)) and TM waves (Figure 6(d)), respectively. For the incident angle from 0° to 60°, the absorptivity of a broad frequency band in 28.88 THz–36.56 THz is higher than 80 % under the illumination of TE polarizations. In terms of the TM waves, the absorptivity is greater than 80 % in the frequency band of 32 THz–37.58 THZ up to the incident angle of 60°. The results indicate a high robustness of the metasurface for the incident angle. Additionally, the absorber we designed is insensitive for both polarization and incident angle in the frequency band of 32 THz–37.58 THz. The broadband characteristics and good absorption performance make the metasurface promising for a mid-infrared absorber.

## 3 Conclusions

In summary, we designed and demonstrated a metasurface integrating multiple distinct functions, according to the dynamic manipulation of multiple DOFs based on phase-change non-local metasurfaces. In the temperature range from room temperature to 53 °C (the insulator state of VO_2_), a dynamic meta-lens is achieved to focus the converted circularly polarized (CP) light at distinct resonant frequencies of quasi-BIC. Simultaneously, the sharp Fano resonance of quasi-BIC enables the metasurface to serve as an optical sensor in the mid-infrared band, yielding a sensitivity of 7.96 THz/RIU at room temperature. Under different temperatures (from room temperature to 53 °C), the sensitivity of the designed metasurface is relatively steady. Furthermore, the designed metasurface converts into a mid-infrared broadband absorber at 80 °C (the metallic state of VO_2_), achieving higher than 80 % absorptivity and an average absorption of 90 % from 28.62 THz to 37.56 THz. The proposed metasurface can effectively improve the availability of devices and be promising to find more new and complex scenarios in sensing, imaging, and communications.

## Supplementary Material

Supplementary Material Details
