# Supplementary material for: Dynamic nonlocal metasurface for multifunctional integration via phase-change materials
Source: Nanophotonics. 2024 Oct 10;13(23):4317–25. doi: 10.1515/nanoph-2024-0357 (PMC11636466; doi:10.1515/nanoph-2024-0357)
Supplement: Supplementary file 1 — Supplementary Material Details [file j_nanoph-2024-0357_suppl_001.docx]

Supplemental Material: Dynamic nonlocal metasurface for multifunctional integration via phase-change materials

Shilin Yu^1,2,3^, Mingfeng Xu^1,2,3,4^, Mingbo Pu^1,2,3,4^*, Xi Tang^1,2,3^, Yuhan Zheng^1,2,3,4^, Yinghui Guo^1,2,3,4^, Fei Zhang^1,2,3,4^, Xiong Li^1,2,4^, Xiaoliang Ma^1,2,4^, and Xiangang Luo^1,2,3,4^*

^1^National Key Laboratory of Optical Field Manipulation Science and Technology, Chinese Academy of Sciences, Chengdu 610209, China;

^2^State Key Laboratory of Optical Technologies on Nano-Fabrication and Micro-Engineering, Institute of Optics and Electronics, Chinese Academy of Sciences, Chengdu 610209, China;

^3^Research Center on Vector Optical Fields, Institute of Optics and Electronics, Chinese Academy of Sciences, Chengdu 610209, China;

^4^College of Materials Science and Opto-Electronic Technology, University of Chinese Academy of Sciences, Beijing 100049, China

E-mail: pmb@ioe.ac.cn, lxg@ioe.ac.cn

In this Supplemental Material, we first describe the simulation method and material properties of the designed metasurface. Lumerical FDTD component of the commercial simulation software Lumerical 2020 R2.4 is employed throughout the paper, including the design of meta-units and the full-mode simulation of the proposed multifunctional metasurface. Fig. S1 displays the permittivity of VO_2_ as a function of frequency under different temperatures, where the data are from experimental measurements of VO_2_ thin film [1]. Next, we investigated the evolution process of quasi-BIC with different values of perturbation at 30 ℃, as shown in Fig. S2. The value of W = 0.6 μm is constant. One can observe that the change values of perturbation can effectively manipulate the quasi-BIC. To further understand the underlying mechanism of the obtained BIC (L = 2 μm), we displayed the magnetic field distributions at the resonance quasi-BIC (43.8 THz) in Fig. S2(c). The near-field distributions are completely consistent with the description in the ref. [2]. Thus, the obtained quasi-BIC is from the space group *P*2 [2,3], and stable when the rectangular apertures rotate within the plane.

The designed metalens can effectively focus the converted RCP light and Fig. S3 describes the transmission spectra of the converted RCP light with different temperatures. Results show the intensity of transmitted RCP light decreases gradually with increasing temperatures, which essentially stems from the higher and higher material loss of VO_2_. Meanwhile, the intensity of transmitted RCP light describes the focusing efficiency of the designed metalens. The efficiencies of the metalens are 6%, 6%, 5%, and 2.6%, corresponding to 30 °C, 40 °C, 45 °C, and 53 °C, respectively. The low efficiencies are attributed to a 25% transmission limitation of cross-conversed light [2,4] and material loss.

To demonstrate the frequency selectivity of the designed metalens, we explored the focus with different frequencies around the quasi-BIC at 30 ℃. The transverse and longitudinal far-field profiles through the focusing spot of the metalens at 30 ℃ for the converted RCP component are shown in Fig. S4 with the corresponding frequencies, 43.86 THz, 43.5 THz, 43.2 THz, and 42.9 THz, respectively. Away from the resonance center, the focused intensity of RCP light decreases gradually and can be ignored at the frequencies 43.2 THz and 42.9 THz, which demonstrates the frequency selectivity. Additionally, we investigate the absorption performance with different temperatures in Fig. S5. Results show that the performance of the available absorption part is almost the same at normal incidence of TE and TM light waves. For the metal state of VO_2_, the absorption performance of the designed metasurface is independent of temperature.


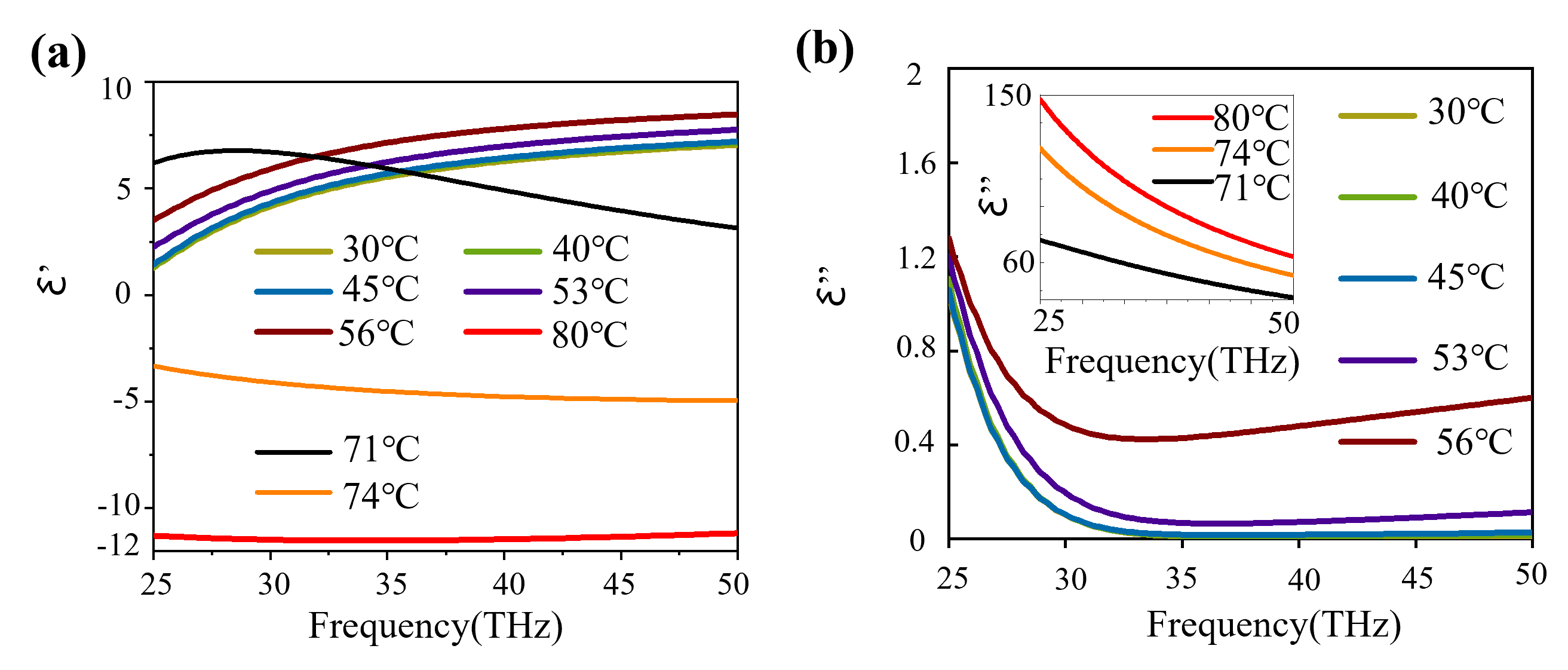


**Fig. S1**: The permittivity of VO_2_ as a function of frequency under different temperatures [1]. (a) Real parts of the permittivity under different temperatures. (b) Imaginary parts of the permittivity under different temperatures.


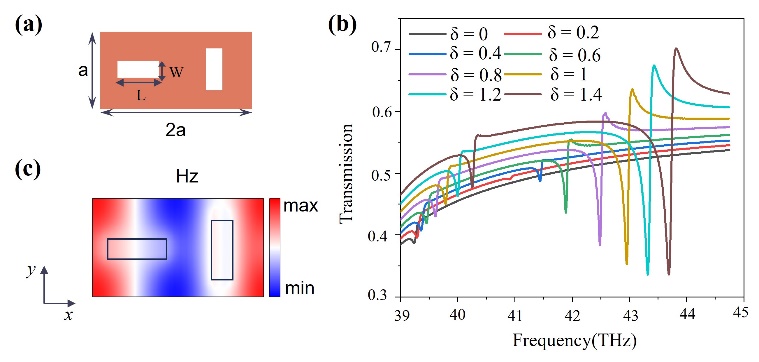


**Fig. S2**: (a) The dimerized meta-atom with rectangular apertures (L = 2 μm, W = 0.6 μm) etched in a VO_2_ thin film, where the period a = 2.5 μm, the thickness t=0.8 μm. (b) The transmission spectra of quasi-BIC with different values of perturbation, wherein δ = L-W. (c) The calculated magnetic field profiles Hz of the quasi-BIC mode, wherein the black rectangles represent the etched rectangular apertures.

**Fig. S3**: The transmission spectra of the converted RCP light with different temperatures.

**Fig. S4**: The transverse and longitudinal far-field profiles through the focusing spot of the metalens at 30 ℃ for the converted RCP component at the corresponding frequencies, 43.86 THz, 43.5 THz, 43.2 THz, and 42.9 THz. Here, the intensity of the focused fields at the frequencies 43.2 THz and 42.9 THz is magnified 10 times and 50 times, respectively.


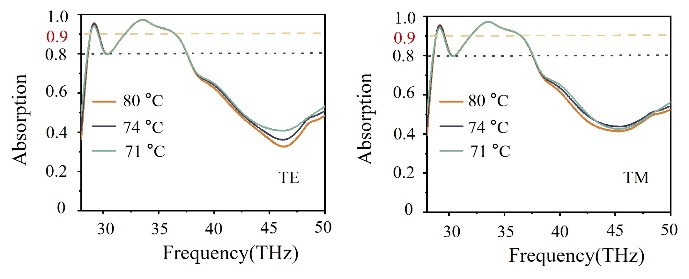


**Fig. S5**: The optical absorption spectra of the designed metasurface for the normal incident TE and TM waves with different temperatures.

References

1. T. Kang, “Research on reconfigurable metasurfaces based on vo2 phase change thin films,” Ph. D. thesis, Univ. Electron. Sci. Tech. China, Chengdu, China, 2023.
2. A. C. Overvig, S. C. Malek, and N. Yu, "Multifunctional nonlocal metasurfaces," *Phys. Rev. Lett.*, vol. 125, no.1, pp. 017402, 2020, <https://doi.org/10.1103/PhysRevLett.125.017402>.
3. A. C. Overvig, S. C. Malek, M.l J. Carter, S. Shrestha, and N. Yu, “Selection rules for quasibound states in the continuum,” *Phys. Rev.B,* vol. 102, pp. 035434, 2020, https://doi.org/10.1103/PhysRevB.102.035434.
4. S. C. Malek, A. C. Overvig, A. Alù, and N. Yu, "Multifunctional resonant wavefront-shaping meta-optics based on multilayer and multi-perturbation nonlocal metasurfaces," *Light Sci. Appl.*, vol. 11, pp. 246, 2022, https://doi.org/10.1038/s41377-022-00905-6.
